# POIROT: a powerful test for parent-of-origin effects in unrelated samples leveraging multiple phenotypes

**DOI:** 10.1093/bioinformatics/btad199

**Published:** 2023-04-17

**Authors:** S Taylor Head, Elizabeth J Leslie, David J Cutler, Michael P Epstein

**Affiliations:** Department of Biostatistics and Bioinformatics, Rollins School of Public Health, Emory University, Atlanta, GA 30322, United States; Department of Human Genetics, Emory University School of Medicine, Atlanta, GA 30322, United States; Department of Human Genetics, Emory University School of Medicine, Atlanta, GA 30322, United States; Department of Human Genetics, Emory University School of Medicine, Atlanta, GA 30322, United States

## Abstract

**Motivation:**

There is widespread interest in identifying genetic variants that exhibit parent-of-origin effects (POEs) wherein the effect of an allele on phenotype expression depends on its parental origin. POEs can arise from different phenomena including genomic imprinting and have been documented for many complex traits. Traditional tests for POEs require family data to determine parental origins of transmitted alleles. As most genome-wide association studies (GWAS) sample unrelated individuals (where allelic parental origin is unknown), the study of POEs in such datasets requires sophisticated statistical methods that exploit genetic patterns we anticipate observing when POEs exist. We propose a method to improve discovery of POE variants in large-scale GWAS samples that leverages potential pleiotropy among multiple correlated traits often collected in such studies. Our method compares the phenotypic covariance matrix of heterozygotes to homozygotes based on a Robust Omnibus Test. We refer to our method as the Parent of Origin Inference using Robust Omnibus Test (POIROT) of multiple quantitative traits.

**Results:**

Through simulation studies, we compared POIROT to a competing univariate variance-based method which considers separate analysis of each phenotype. We observed POIROT to be well-calibrated with improved power to detect POEs compared to univariate methods. POIROT is robust to non-normality of phenotypes and can adjust for population stratification and other confounders. Finally, we applied POIROT to GWAS data from the UK Biobank using BMI and two cholesterol phenotypes. We identified 338 genome-wide significant loci for follow-up investigation.

**Availability and implementation:**

The code for this method is available at https://github.com/staylorhead/POIROT-POE.

## 1 Introduction

Most genome-wide association studies (GWAS) implicitly assume the magnitude and direction of effect of a genetic variant on expression of a phenotype is independent of whether the variant was maternally or paternally inherited. However, there exists a distinct class of genetic variants for which this assumption is violated. Such variants harbor a parent-of-origin effect (POE) wherein the effect of an allele on a trait depends on whether it was transmitted from the mother or the father (Lawson et al. 2013). A substantial proportion of the variation in complex traits is not explained by the additive effects of common single-nucleotide polymorphisms (SNPs) across the genome. POEs may represent an important contribution to this missing heritability (Guilmatre and Sharp 2012).

There are multiple cited biological mechanisms by which POEs can arise in mammals. These include maternal intrauterine environment effects and effects of the maternal mitochondrial genome. However, the most frequently considered mechanism is genomic imprinting (Rampersaud et al. 2008). This epigenetic phenomenon was formally discovered in the 1980s primarily through embryological experiments (Reik and Walter 2001). In imprinting, the maternal and paternal alleles undergo differential epigenetic modifications that leads to parent-of-origin-specific transcription of the gene copies. Many imprinted genes tend to be found in clusters across the genome. Regulation of the expression of these clustered genes is under control of an imprinting control region (ICR), the mechanisms of which are complex (Barlow 2011). These ICR are often characterized by repetitive sequences and located near imprinted genes. It is estimated that only ∼1% of mammalian genes are subject to imprinting. However, there has been growing evidence for the existence of POE variants for a wide range of hereditary traits (Peters 2014). Classic examples of POE-associated diseases include Prader–Willi syndrome and Angelman syndrome. These diseases result from imprinted genes at 15q11-15q13 when only maternal or paternal copies are expressed, respectively (Aypar et al. 2014). Considerable research has further suggested POEs originate for a wide spectrum of complex traits, including obesity-related phenotypes, type 2 diabetes, basal-cell carcinoma, attention-deficit/hyperactivity disorder, schizophrenia, and breast cancer (Giannoukakis et al. 1993; Temple et al. 1995; Huxtable et al. 2000; Polychronakos and Kukuvitis, 2002; Dong et al. 2005; Palmer et al. 2006; Rampersaud et al. 2008; Kong et al. 2009; Wang et al. 2012; Hoggart et al. 2014).

To detect variants demonstrating POEs, studies have historically required genotype data from related individuals to ascertain parental ancestry of the inherited alleles. In the case of available parent-offspring trio or other forms of familial genomes, there are well-established methods to detect POEs (Weinberg et al. 1998; Weinberg 1999; Sinsheimer et al. 2003; Cordell et al. 2004; Becker et al. 2006; Ainsworth et al. 2011; Howey and Cordell 2012; Zhou et al. 2012; Connolly and Heron 2015; Howey et al. 2015). These approaches often test for a mean difference in allele effect based on grouping offspring by parent-of-origin of the allele. These mean-based tests are intuitive and optimally powered given sample size. Yet, the requirement of trio or more general family data severely limits this sample size in practice. This, in consequence, limits genome-wide discovery of the modest genetic effects that we anticipate for complex human traits.

Rather than rely on family studies of limited sample size to detect POEs, researchers have recently transitioned to detecting the phenomenon in GWAS-scale cohorts. This practice requires innovative statistical methods to deal with missing parental ancestry information. For example, Kong et al. (2009) inferred parental origin of alleles when parental genotype data are not available by first phasing Icelandic probands. For each of the proband haplotypes, they searched a genealogy database for the closest typed maternal and paternal relatives carrying that haplotype (Kong et al. 2009). For each haplotype, they constructed a robust score comparing the meiotic distances between the proband and these two relatives to quantify the likelihood of maternal or paternal transmission and ultimately assign parental origin. While this approach solves the issue of small sample sizes, power is still impacted by the potential inaccuracy or uncertainty in haplotypic reconstruction.

More recently, Hoggart et al. (2014) described a novel statistical method for detecting POEs for a single quantitative trait using GWAS data of unrelated individuals. The authors illustrated that the existence of a POE results in increased phenotypic variance among heterozygotes compared to homozygotes. They tested for this variance inflation using a robust version of the Brown–Forsythe test. The method successfully identified previously undocumented POE associations of two SNPs with body mass index (BMI). This work has enabled POE analysis in population studies of biobank scale.

A sizable proportion of genes in the GWAS catalog are pleiotropic (Chesmore et al. 2018). These genes affect more than one biological process, in turn associating with multiple (correlated) phenotypes (He and Zhang 2006). Analyzing the joint effects of a gene on more than one trait can often result in a marked increase in power over univariate approaches (O’Reilly et al. 2012; Solovieff et al. 2013; Kocarnik and Fullerton 2014). Importantly, well-established POEs in humans are usually the result of embryonic silencing of one parental allele. As this silencing generally occurs early in development, its effects are likely to present in all or nearly all tissues expressing the gene. When differential silencing of this gene affects multiple tissues, this can yield POEs for several distinct phenotypes. Joint analysis of multiple traits can leverage this potential pleiotropy to improve power over univariate variance-based POE tests while simultaneously reducing multiple testing burden of multiple phenotypes.

Here, we expand on the concept initially suggested by Hoggart et al. to develop a test for POEs in genetic studies of unrelated individuals that considers multiple quantitative phenotypes. We show that a pleiotropic POE variant will not only induce differences in the variance of POE traits between heterozygotes and homozygotes, but also in their corresponding covariances. In our method, POIROT (Parent-of-Origin Inference using Robust Omnibus Test), we test for equality of phenotypic covariances matrices between heterozygous and homozygous groups. Specifically, we use the robust omnibus (R-Omnibus) test (O’Brien 1992) to accommodate highly skewed traits. We first provide background on the statistical construction of our test statistic using the R-Omnibus framework. Next, through simulations, we demonstrate that our proposed method properly controls type I error and achieves superior power compared to the univariate approach of Hoggart et al. We also introduce a *post hoc* test that can help distinguish variants with POE effects from variants demonstrating more general gene–gene/gene–environment effects (which also induce patterns of trait variance/covariance that differ by genotype). We apply our method to GWAS data of BMI, high-density lipoprotein (HDL) cholesterol, and low-density lipoprotein (LDL) cholesterol from the UK Biobank and identify 338 significant potential POE loci. We conclude with a discussion of our findings, limitations, and proposed research to extend this work.

## 2 Materials and methods

### 2.1 Phenotype model

Using the notation of Hoggart et al. (2014) consider one biallelic SNP with reference allele A and alternative allele B. Assume we have collected $n_{\mathrm{AA}}$ individuals who have the homozygous AA genotype, $n_{\mathrm{BB}}$ individuals who have the homozygous BB genotype, and $n_{AB}$ individuals who are heterozygous. Further assume we have collected *K *>* *1 continuous phenotypes on all subjects and that we have already adjusted these phenotypes for the effects of non-genetic confounders like principal components of ancestry.

We first model phenotypes in homozygous AA subjects. Let $y_{i}^{\left(\mathrm{AA}\right)}={\left(y_{i,1}^{\left(\mathrm{AA}\right)},y_{i,2}^{\left(\mathrm{AA}\right)},\ldots ,y_{i,K}^{\left(\mathrm{AA}\right)}\right)}^{\prime }\in R^{K}\;$be the vector of phenotypes for the *i*th AA individual. We can represent $y_{i}^{\left(\mathrm{AA}\right)}$ using the following framework


(1) yiAA=μ+ϵi,i=1,…,nAA

Within (1), $\mu =(\mu_{1},\ldots ,\mu_{K})\prime$ is the K×1 vector of phenotype means in AA subjects and $\epsilon_{i}=(\epsilon_{i1},\ldots ,\epsilon_{iK})\prime$ is the K×1 vector of error terms. We assume that $E\left[\epsilon_{i}\right]=0_{K}$ and $\text{Cov}\left[\epsilon_{i}\right]=\Sigma$, where Σ is the K×K variance–covariance matrix of the vector of error terms.

We next model phenotypes in homozygous BB subjects. Let $y_{i}^{\left(\mathrm{BB}\right)}={\left(y_{i,1}^{\left(\mathrm{BB}\right)},y_{i,2}^{\left(\mathrm{BB}\right)},\ldots ,y_{i,K}^{\left(\mathrm{BB}\right)}\right)}^{\prime }\in R^{K}$ be the vector of phenotypes for the *i*th BB individual. Further, let $\beta_{Mk}$ and $\beta_{Pk}$ represent the effect of the maternally inherited and paternally inherited B allele, respectively, on the *k*th phenotype. If there is no association between this SNP and the *k*th phenotype, it follows that $\beta_{Mk}=\beta_{Pk}=0$. If there is a marginal association between this SNP and the *k*th phenotype, but there is no POE present, then $\beta_{Mk}=\beta_{Pk}\neq 0$. With this notation defined, we can model $y_{i}^{\left(\mathrm{BB}\right)}$ as


(2) yiBB=μ+βM+βP+ϵi,i=1,…,nBB

Here, μ is as defined previously for (1), $\beta_{M}=(\beta_{M1},\ldots ,\beta_{MK})\prime$ is the K×1 vector of maternal effects of the B allele on each of the *k* phenotypes, and $\beta_{P}=(\beta_{P1},\ldots ,\beta_{PK})\prime$ is the K×1 vector of corresponding paternal effects of the B allele. Each element of $\beta_{M}$ and $\beta_{P}\;$ is assumed to be a fixed effect. Just as for the AA subjects in (1), we assume that $E\left[\epsilon_{i}\right]=0_{K}$ and $\text{Cov}\left[\epsilon_{i}\right]=\Sigma$.

Lastly, we consider heterozygous AB individuals who carry only one copy of the alternative allele B. Let $y_{i}^{\left(\mathrm{AB}\right)}={\left(y_{i,1}^{\left(\mathrm{AB}\right)},y_{i,2}^{\left(\mathrm{AB}\right)},\ldots ,y_{i,K}^{\left(\mathrm{AB}\right)}\right)}^{\prime }\in R^{K}$ be the vector of phenotypes for the *i*th heterozygote. We can model this vector as


(3) yiAB=μ+πiβM+1-πiβP+ϵi,i=1,…,nAB.

In (3), $\pi_{i}$ is an indicator random variable where $\pi_{i}=1$ if individual *i* received the B allele from the mother and $\pi_{i}=0$ if individual *i* received the B allele from the father. We assume $\pi_{i}\;∼$ Bernoulli (½), as we expect that half of heterozygotes will have maternally-derived B alleles. The maternal and paternal effect vectors are as defined as for the model of BB subjects. We also assume that $E\left[\epsilon_{i}\right]=0_{K}$ and $\text{Cov}\left[\epsilon_{i}\right]=\Sigma$. In other words, the covariance matrix of the error terms is the same within all three genotype groups.

Based on the derivations above, we can calculate the phenotype covariance matrix for each genotype category. Based on Equations (1) and (2), it is straightforward to show that the phenotype covariance matrix of AA individuals (Σ) is the same as the analogous matrix for BB individuals. Therefore, we can define $\Sigma_{\mathrm{Hom}}=\Sigma$ as the phenotypic covariance matrix for all homozygous subjects. For heterozygous AB subjects modeled in Equation (3), we can show that (assuming $\pi_{i}\;⊥\;\epsilon_{i}\;\forall \;i,i\in \left(1,\ldots ,n_{\mathrm{AB}}\right)$) the phenotype covariance matrix for heterozygotes is $\Sigma_{\mathrm{Het}}=\frac{1}{4}\left(\beta_{M}-\beta_{P}\right){\left(\beta_{M}-\beta_{P}\right)}^{\prime }+\Sigma_{\mathrm{Hom}}$. Defining $b_{k}=\beta_{Mk}-\beta_{Pk}\;\left(k=1,\ldots ,K\right)$, we can show that ${\Sigma_{\mathrm{Het}}=\Sigma }_{\mathrm{Hom}}$ if and only if


(4) b12b1b2⋯b1bKb2b1b22…b2bK⋮⋮⋱⋮bKb1bKb2⋯bK2=0K×K 

This observation motivates the use of a test of equality of two covariance matrices for detecting POEs in a population-based sample where we cannot explicitly observe $\pi_{i}$. If a POE SNP exists for any phenotype *k*, then $b_{k}\neq 0$ and $b_{k}^{2}>0$. Thus, the *k*th diagonal element of $\Sigma_{\mathrm{Het}}$ will be larger than the corresponding element of $\Sigma_{\mathrm{Hom}}$. Furthermore, if the SNP has POEs on two phenotypes *k* and *k*’, then $b_{k}b_{k^{\prime }}\neq 0$. The *kk’* element of $\Sigma_{\mathrm{Het}}$ will also be different from the corresponding off-diagonal element of $\Sigma_{\mathrm{Hom}}$.

### 2.2 POIROT method to detect POE SNPs

We can test the null hypothesis that no POEs exist at a given SNP for any of the K phenotypes under study ($H_{0}:\;\beta_{M}=\beta_{P}$) by equivalently testing $H_{0}:\;\Sigma_{\mathrm{Het}}=\Sigma_{\mathrm{Hom}}$. In our proposed method POIROT, we test for equality of these phenotypic covariance matrices between homozygotes and heterozygotes using the robust omnibus (R-Omnibus) test of O’Brien (O’Brien 1992). POIROT uses R-Omnibus rather than the traditional Box’s M test (Box 1949) to test covariance differences since the latter is highly sensitive to deviations of phenotypes from multivariate normality. This can lead to a undesirable elevation in type I error rates (Tiku and Balakrishnan 1984).

To derive the R-Omnibus test, we first center the phenotypes by the median within each genotype group (AA, AB, BB). This step is necessary if a marginal association exists between the alternative allele and a given phenotype. In that event, the variance of original phenotype values among aggregate homozygous subjects (AA, BB) would be erroneously inflated. We next group these centered phenotypes by homozygote versus heterozygote status. Let $x_{i,k}^{\mathrm{het}}$ be the *k*th centered phenotype of the *i*th heterozygote ($i=1,\ldots ,n_{\mathrm{AB}})$ and $x_{j,k}^{\mathrm{hom}}\;$be the *k*th phenotype of the *j*th homozygous (AA and BB combined) individual ($j=1,\ldots ,n_{\mathrm{AA}}+n_{\mathrm{BB}}$). We then calculate the median of each phenotype *k* in heterozygotes and homozygotes separately. Let $M_{k}^{\mathrm{het}}$ be the median of the *k*th phenotype in the $n_{\mathrm{AB}}$ heterozygotes. Correspondingly, let$\;M_{k}^{\mathrm{hom}}$ be the median of the *k*th phenotype in the $n_{\mathrm{AA}}$ + $n_{\mathrm{BB}}$ homozygotes. For heterozygotes and homozygotes separately, we then calculate for phenotypes *k* and *k’*:


(5) Zi,k,k'het=xi,khet-Mkhetxi,k'het-Mk'het

(6) Zj,k,k'hom=xj,khom-Mkhomxj,k'hom-Mk'hom

(7) $$\begin{array}{c} W_{i,k,k^{\prime }}^{\mathrm{het}}=\frac{Z_{i,k,k^{\prime }}^{\mathrm{het}}}{{\left|Z_{i,k,k^{\prime }}^{\mathrm{het}}\right|}^{\frac{1}{2}}} \end{array}$$

(8) Wj,k,k'hom=Zj,k,k'homZj,k,k'hom12

In (7) and (8), we standardize the *Z* measures by dividing by the square root of their absolute values. We consider $W_{i}^{\mathrm{het}}$ to be the vector of *W* values for the *i*th heterozygous subject, and $W_{j}^{\mathrm{hom}}$ is the corresponding vector of *W* values for the *j*th homozygous subject. We then perform a two-sample Hotelling’s T^2^ test (Hotelling 1931) comparing our two sets of $p=(K^{2}+K)/2$ sample means (${\bar{W}}_{\mathrm{het}},\;{\bar{W}}_{\mathrm{hom}})$. There are p dependent variables being compared between heterozygotes and homozygotes as this corresponds to the number of upper-triangular elements in the phenotypic covariance matrix. We calculate the test statistic $t^{2}=\frac{n_{\mathrm{het}}n_{\mathrm{hom}}}{n_{\mathrm{het}}+n_{\mathrm{hom}}}\left({\bar{W}}_{\mathrm{het}}-{\bar{W}}_{\mathrm{hom}}\right)\prime S^{-1}({\bar{W}}_{\mathrm{het}}-{\bar{W}}_{\mathrm{hom}})$, where $S^{-1}$ is the inverse of the pooled covariance matrix estimate. Under the null, our test statistic $t^{2}∼T^{2}\left(p,n_{\mathrm{het}}+n_{\mathrm{hom}}-2\right)$ (Hotelling 1931). The test can also be viewed as a one-way multivariate analysis of variance test (MANOVA).

### 2.3 *Post hoc* test for interaction effects

As detailed above, POIROT tests for a variant demonstrating POE by comparing/contrasting trait variances and covariances by genotype. However, trait variances can also differ by genotype when a variant exhibits a gene–gene (GxG) or gene–environment (GxE) interaction effect (Paré et al. 2010). To increase confidence that a variant identified by POIROT demonstrates a POE rather than a more general interaction effect, we propose a *post hoc* test that can be utilized to differentiate the two phenomena. The test is motivated by the observation that, for a general interaction effect, the variance of a quantitative phenotype among BB homozygous individuals is different from that of AA homozygotes. This observation is in contrast to the variance pattern expected under a POE, in which the variability of each homozygous group is the same after phenotype centering. Thus, we can craft a *post hoc* test that assesses the null hypothesis of a POE (trait variance/covariances are the same between the two homozygous categories) versus the alternative of a general interaction effect (trait variance/covariances differ between the two homozygous categories). We create such a test by implementing the R-Omnibus framework as previously outlined in Section 2.2 but restricted to comparison of the two homozygous groups (AA, BB).

### 2.4. Simulation study

We conducted a variety of simulation studies to determine POIROT’s ability to detect POEs while maintaining proper rates of type I error. We considered *K *=* *3, 6, or 10 phenotypes and *n *=* *3000, 5000, or 10 000 unrelated individuals. To generate phenotypes for each round of simulation, we first randomly generate *K* intercepts from a standard normal distribution to form the K×1 vector μ. This corresponds to the mean vector of phenotypes among AA homozygotes. For simplicity, we assume the diagonal elements of the matrix Σ, corresponding to the variances of the random error terms, are all equal to one. We assume the *K* phenotypes exhibit one of three possible levels of pairwise correlation (low, medium, or high). We assume the pairwise trait correlations are randomly drawn from a uniform distribution. To simulate phenotypes exhibiting “low” correlation, we assume this is a Uniform(0,0.3) distribution. For phenotypes of “medium” and “high” correlation, we assume a Uniform(0.3,0.5) and Uniform(0.5,0.7) distribution, respectively. These random draws are used to populate the off-diagonal elements of Σ.

Once we have constructed Σ, we then randomly generate *n* maternal and paternal genotypes for a given SNP by sampling twice from a Bernoulli [*p* = MAF (minor allele frequency)] distribution for each parent. To generate offspring genotypes, we sample from a Bernoulli (*p *=* *.5) distribution to determine which maternal allele and which paternal allele is transmitted. Thus, we can now assign all *n* offspring to one of four genotype groups: (i) AB with maternal reference/paternal alternative, (ii) AB with paternal reference/maternal alternative, (iii) AA, and (iv) BB. We then simulate the phenotypic error vector for all *n* unrelated offspring by drawing from a multivariate distribution with mean 0 and variance-covariance matrix Σ. The respective fixed K×1 maternal and paternal effect vectors of the alternative allele ($\beta_{M},\beta_{P}$) are constructed depending on the specific null or alternative scenario under consideration. We then add these vectors to the random error and intercept term in concordance with the genotype group of each individual, as described in Section 2.1.

For type I error rate simulations, as described above, we assume these phenotypes have pairwise-trait correlation of levels low, medium, or high. To reflect the scenario where there exist no POEs or marginal effects of the alternative allele at the locus for any phenotype, we assume that $\beta_{M}=\beta_{P}=0$. We also considered a second null scenario wherein a marginal association exists for the variant that is not specific to the parent of origin, i.e. $\beta_{M}=\beta_{P}\neq 0$. However, we note that if the same seeds are used in simulating the data, this marginal fixed effect is effectively removed when centering phenotypes by genotype group. The resulting test statistics are equivalent to the first null scenario. We first consider the circumstance where the random error terms are drawn from a normal distribution, i.e. the error follows ${\mathrm{MVN}}_{K}(0,\Sigma )$ and assume a MAF of 0.25. For each of the 27 combinations of number of phenotypes, sample size, and pairwise-trait correlation, we conducted 50 000 null simulations. To evaluate the robustness of our method to highly skewed phenotypes, we then repeated these parameter settings with non-normal random error terms. In particular, we utilize the method of Vale and Maurelli to simulate multivariate non-normal error terms assuming a skewness of two and excess kurtosis of two for each phenotype (Vale and Maurelli 1983). An example distribution of such a phenotype is illustrated in Supplementary Fig. S1.

Next, we investigated the power of our test when POEs do in fact exist for a locus. We again considered *K* = 3, 6, or 10 normally distributed phenotypes. We assumed 1, 2, or 3 had parent-of-origin specific associations with the variant. When the number of affected phenotypes is greater than one, this corresponds to pleiotropy. For these scenarios, we assumed $\beta_{P}=0$ and $\beta_{Mk}$ = 0.5, 0.6, or 0.75 for each phenotype *k* harboring a POE. All other elements of the maternal effect vector are 0 for the phenotypes with no POE associations. We again considered low, medium, and high pairwise-trait correlations. We assumed a MAF of 0.25 and sample sizes of 5000, and 10 000. We applied our method to 5000 simulated datasets for each of the 162 settings and calculated power at significance level $\alpha \in \{0.005,\;5\;{\times \;10}^{-4}\}$. We also evaluated the power of POIROT when a locus is pleiotropic for POEs, but the magnitude of $\beta_{Mk}$ varies by phenotype. For this power analysis, we again tested 3, 6, or 10 total normal phenotypes, of which 2 or 3 are harboring POEs. Since maternal effect sizes of 0.5–0.75 were considered for the scenarios described above, we tested $\beta_{M1}=0.5$, $\beta_{M2}=0.75$ when two phenotypes have POEs. When three phenotypes have POEs, we tested power using 0.5, 0.6, and 0.75 as maternal effect sizes.

We also compared the performance of POIROT to the corresponding univariate test of Hoggart et al. (2014). For the univariate test, we first calculated power using standard Bonferroni correction. Power was calculated as the proportion of loci for which the minimum observed *P*-value across the *K* phenotypes tested was less than α/K. Given that these phenotypes are correlated and therefore may not reflect *K* independent tests, this approach can be overly conservative. Thus, we implemented a second more liberal approach that estimates the true number of independent tests, $K_{\mathrm{eff}}$, which corresponds to the minimum number of principal components (PCs) explaining 90% of the variation in our *K* phenotypes. We then calculated power of the univariate approach as the proportion of loci for which the minimum observed *P*-value was less than $\alpha /K_{\mathrm{eff}}$ (Gao et al. 2008; Broadaway et al. 2016). We then repeated these parameter settings for assessing power of POIROT with non-normal phenotypes, as described for null simulations.

Finally, we performed several simulations to investigate the performance of our proposed *post hoc* test for distinguishing POEs from general interaction effects. Under the null hypothesis (i.e. there exist POEs but no interaction effects for any of the phenotypes considered), we looked at type I error of the R-Omnibus test comparing phenotypic covariances of the two homozygous groups. Similar to above, we considered a MAF of 0.25 and 3, 6, or 10 tested phenotypes, of which 1, 2, or 3 had POEs but no interaction effects. We considered sample sizes of 5000 and 10 000, maternal POE effect sizes {0.5, 0.6, 0.75}, and low/medium/high trait correlation. We also evaluated the power of this *post hoc* test to identify GxE effects when present. Simulation parameters were informed by prior work of Paré et al. (2010). We considered a single unmeasured covariate drawn from a standard normal distribution. Again, we considered 3, 6, or 10 total quantitative traits, of which three had a non-negligible covariate effect. Of these three phenotypes, 1, 2, or 3 had gene–covariate interaction effects. The covariate effect sizes ranged from 0.3 to 0.7. Among the phenotypes with gene–covariate interaction effects, we varied to the proportion of total variation of each phenotype explained by the interaction effects between 0.005 and 0.01. Again, we allowed traits to have varying pairwise correlation. We performed 5000 simulations for each of the 216 power settings outlined for the *post hoc* interaction test.

### 2.5 Application of POIROT to UK Biobank

To assess the utility of POIROT for detecting POEs on continuous phenotypes using published population-based GWAS data, we utilized genotype and phenotype data from the UK Biobank (UKB), a large-scale biomedical database housing data collected from approximately 500 000 individuals from the UK (see Acknowledgements). This study allows for widespread investigation of the genetic variation associated with hundreds of lifestyle and health factors. To identify potential POE variants, we obtained data on three quantitative phenotypes [BMI (kg/m^2^), HDL cholesterol (mmol/l), and LDL cholesterol (mmol/l)]. Relevant covariates included genotyping array, PCs, sex, age at recruitment, and smoking status (prefer not to answer, never, previous, current). Prior to analysis, we removed all individuals identified as outliers according to pre-calculated metrics of genotype missingness, heterozygosity, and excess relativeness. We excluded those with putative sex chromosome aneuploidy and those who were not included in PCA calculation. We included individuals of self-reported white British ancestry only.

Subjects were genotyped using either the UK BiLEVE or UK Biobank Axiom arrays. We considered only autosomal variants with MAF >0.05, Hardy–Weinberg equilibrium *P *>* *1e−8, and missingness rate <0.02. After quality control and filtering, 330 801 SNPs remained for analysis across 292 779 unrelated individuals with complete phenotype and covariate information. There is moderate negative correlation between BMI and HDL cholesterol (Pearson’s *r* = −0.35), low positive correlation between BMI and LDL (*r* = 0.02), and low positive correlation between LDL and HDL (*r* = 0.10). However, all estimated correlations are statistically significant (*P* < 2.2e−16). Covariate adjustment was performed by first fitting a linear model for each phenotype and extracting the residuals as the new adjusted phenotypes. We then applied POIROT to these three adjusted phenotypes to jointly test for POEs across the genome. We compared the findings of our approach to those from the method of Hoggart et al. performed on each phenotype individually. For any variant identified by POIROT meeting the Bonferroni-adjusted genome-wide significance threshold, we applied our proposed *post hoc* test to assess if the effect might be explained by a general interaction effect rather than a POE.

We concluded with a follow-up analysis to determine whether we see enrichment of variants in imprinting regions among those with lowest POIROT *P*-values for detecting POEs in the UKB cohort. We first downloaded genes of known imprinting and predicted imprinting status in humans from the GeneImprint database (https://www.geneimprint.com/). We then determined which variants in the UKB dataset fell within 500 kb of the starting and ending site of these genes. We defined these as our variant set of interest [comparable to a gene set in Gene Set Enrichment Analysis (GSEA)]. We then utilized the GSEAPreranked tool to test for enrichment of variants in this set among those top ranked variants by -log10(POIROT *P*-value) (Mootha et al. 2003; Subramanian et al. 2005).

## 3 Results

### 3.1 Type I error rate

**Figure 1:**
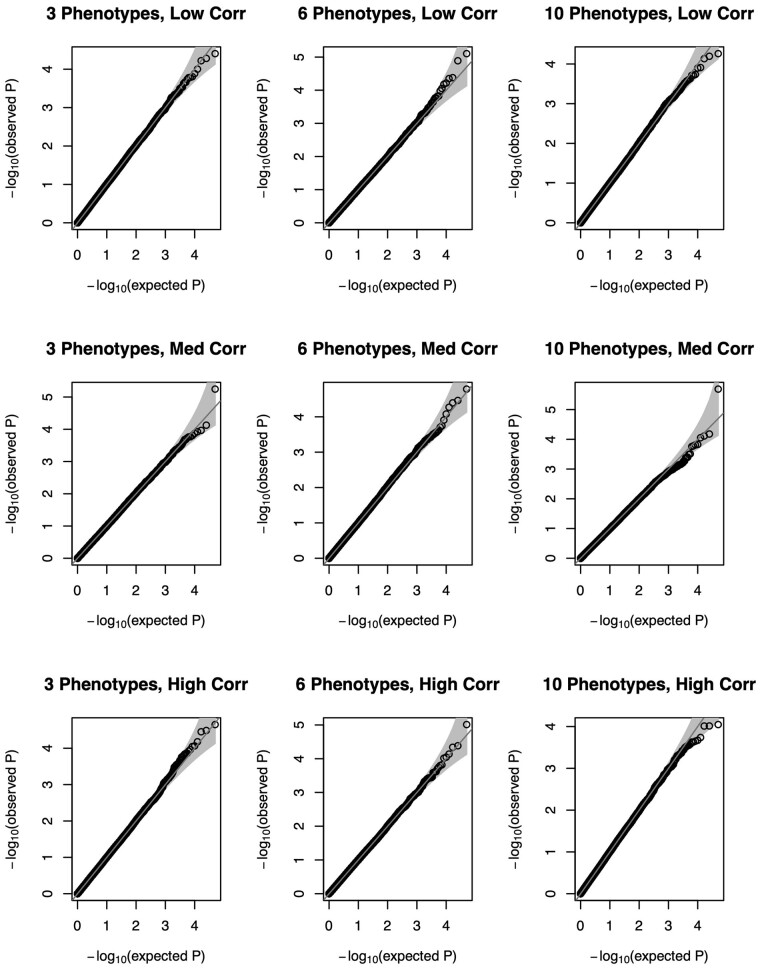
QQ plots of *P*-values for proposed parent-of-origin effect test under the null hypothesis $\beta_{M}=\beta_{P}=0$ using a series of 50 000 simulations of 5000 individuals using 3 (left column), 6 (middle column), or 10 (right column) continuous normal phenotypes. MAF is assumed to be 0.25. Horizontal panels depict level of pairwise-trait correlation (low, medium, high). QQ, quantile–quantile; MAF, minor allele frequency.

**Figure 2:**
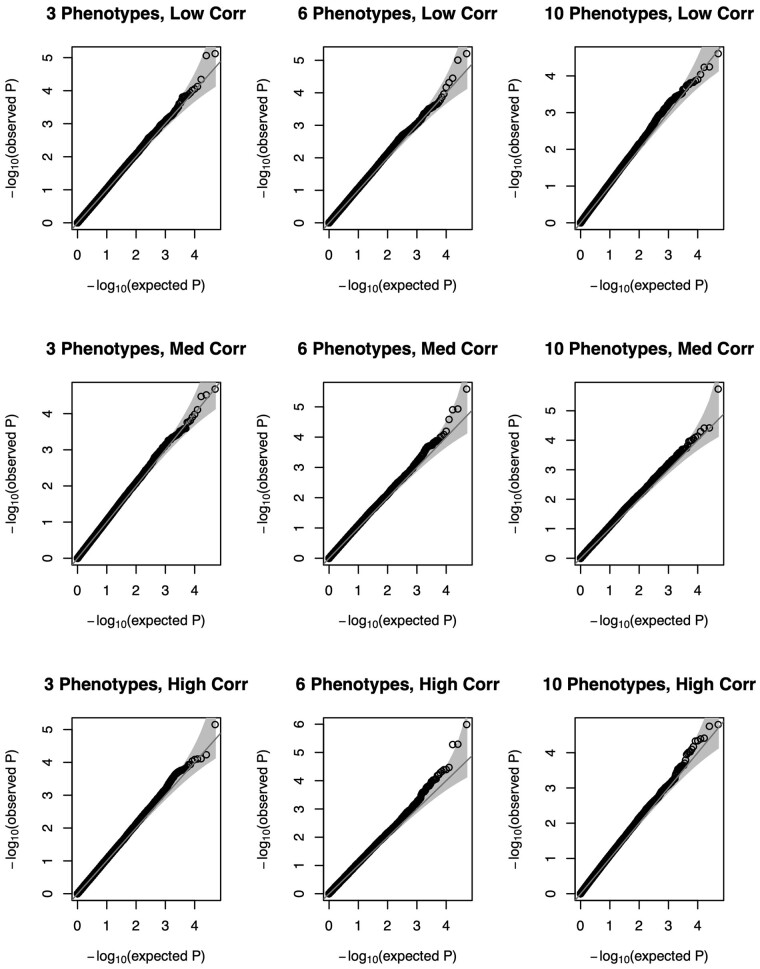
QQ plots of *P*-values for proposed parent-of-origin effect test under the null hypothesis $\beta_{M}=\beta_{P}=0$ using a series of 50 000 simulations of 5000 individuals using 3 (left column), 6 (middle column), or 10 (right column) continuous non-normal phenotypes. MAF is assumed to be 0.25. Horizontal panels depict level of pairwise-trait correlation (low, medium, high). QQ, quantile–quantile; MAF, minor allele frequency.

We summarize the type I error of null scenarios with a sample size of 5000 individuals using Quantile-Quantile (QQ) plots in Fig. 1 (normal traits) and Fig. 2 (non-normal traits). Across the settings considered, our method yields the expected distribution of *P*-values under the null hypothesis of no POEs for any single phenotype. The distribution of the *P*-values is again as expected under the null when we have non-normality of phenotypes (Fig. 2), suggesting our method remains robust. We summarize the empirical type I error rates of our proposed test and the competing univariate approach at significance level $\alpha \in \{0.05,\;0.005,\;5\;{\times \;10}^{-4},5\;{\times \;10}^{-5}\}$ in Supplementary Table S1. POIROT maintained appropriate type I error across all scenarios for normally distributed traits. We observed slightly higher error when 6 or 10 highly-skewed non-normal phenotypes were tested. The univariate approach with correction for $K_{\mathrm{eff}}$ tests showed minor inflation with 6 or 10 highly correlated phenotypes.

### 3.2 Power

**Figure 3:**
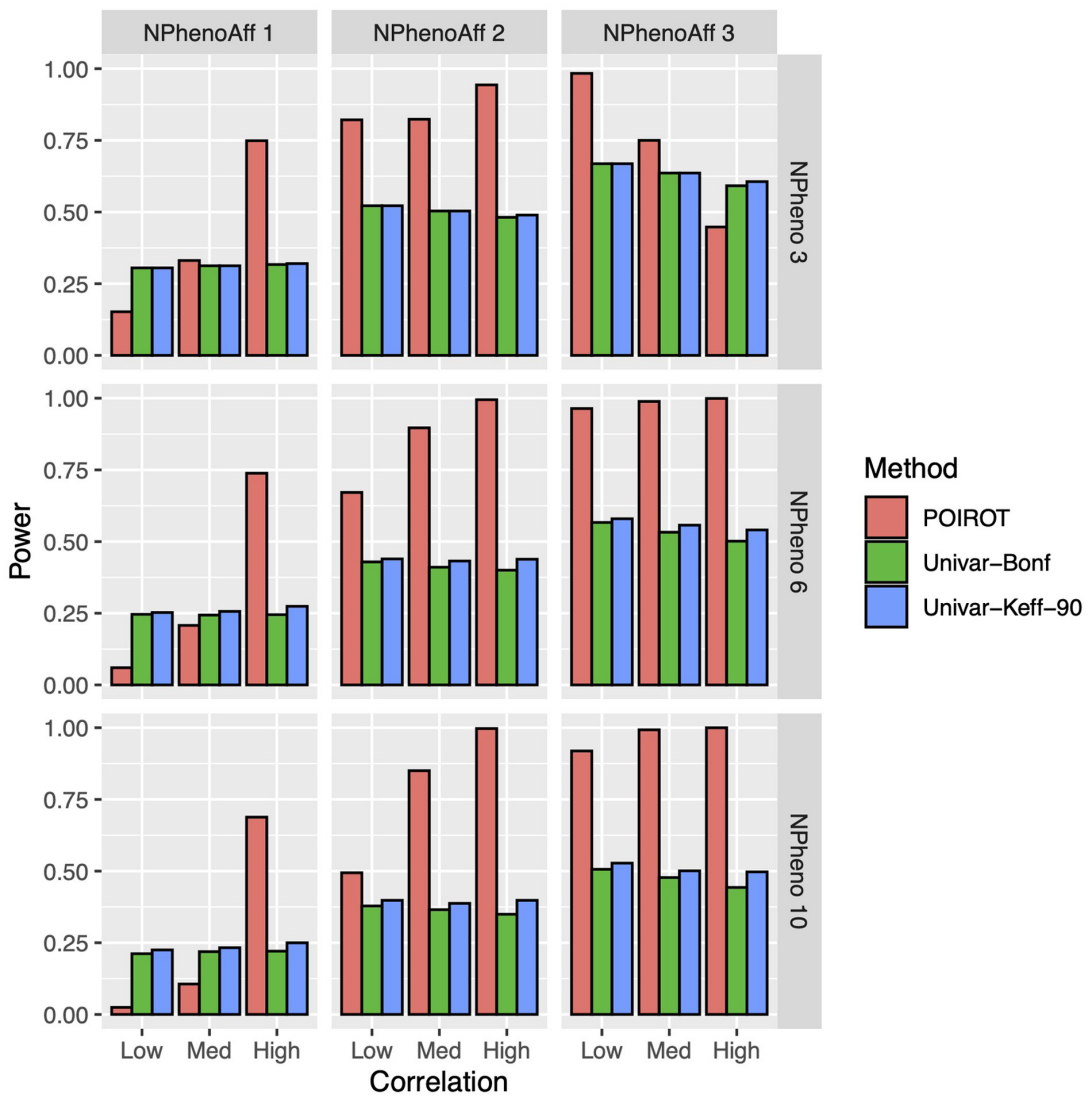
Power of POIROT to identify POEs assuming *K *=* *3, 6, or 10 normal phenotypes (horizontal panels) compared to univariate test. We assume either 1, 2, or 3 of the phenotypes harbor POEs at the locus (vertical panels). We performed 5000 simulations for each scenario. We calculated power at significance level 0.0005 for our multitrait test and 0.0005/*K* (Bonferroni correction) and 0.0005/*K_eff_* for the univariate test, where *K_eff_* is the number of PCs needed to explain 90% phenotypic variation. $\beta_{Mk}=0.75$ for POE traits, MAF = 0.25, and sample size = 5000. POE, parent-of-origin effect; MAF, minor allele frequency; PCs, principal components.

Simulation results comparing the performance of POIROT to the competing univariate test under the assumption of true POE(s) are summarized in Fig. 3. This figure reflects normally distributed traits and sample size of 5000 ($\alpha =\;5\;{\times \;10}^{-4}$). Corresponding results from all other additional power settings, including both normal and non-normal traits, sample sizes of 5000 and 10 000, and $\alpha =\;0.005,\;5{\;\times \;10}^{-4}$ are provided in Supplementary Figs S2–S9.

Simple Bonferroni correction tends to be overly conservative in the presence of correlated traits. We therefore used two multiple-testing correction approaches for the univariate method. As power generally increases with increasing sample size and POE magnitude, the scenarios shown in Fig. 3 correspond to a $\beta_{Mk}$ of 0.75 and sample size of 5000. For almost all scenarios, we see three general trends. First, unlike the univariate method, our method successfully leverages the correlation among phenotypes. We see power increasing with increasing trait correlation. Second, when pleiotropy exists and more than one phenotype harbors a POE, our method outperforms the univariate approach regardless of the multiple testing correction strategy. Third, power of POIROT increases as the number of phenotypes associated with the maternally-transmitted alternative allele increases across all levels of phenotypic correlation. Under simulated pleiotropic POE loci with varying $\beta_{Mk}$, the power of POIROT tends to reflect the power assuming a constant $\beta_{Mk}$ for POE phenotypes at the median effect size (Supplementary Figs S10 and S11).

The one exception to these trends is the top right panel of Fig. 3. This reflects the scenario where three of three phenotypes harbor POEs of the same magnitude and direction. We see here that power decreases going from low to medium correlation and from medium to high correlation. We also see lower power when three phenotypes are affected when compared to the corresponding settings when only two of three phenotypes have POEs. This pattern, although unusual, has been documented in previous cross-phenotype methodological studies (Broadaway et al. 2016; Ray et al. 2016). As described in Section 2.2, the R-Omnibus test for equality of covariance matrices used by POIROT ultimately employs a one-way MANOVA test to generate our test statistic. Ray et al. describe how when we have K correlated traits being tested and a SNP is associated with all K traits, utilizing a MANOVA to find marginal associations with multiple traits can result in an appreciable loss of power. In particular, the authors show how the power of MANOVA is asymptotically lower when all traits are associated with equal magnitude and direction than when fewer than K phenotypes are associated (Ray et al. 2016).

### 3.3 *Post hoc* interaction test

Type I error results of our *post hoc* test for distinguishing POE (null) from general interaction effects (alternative) are shown in Supplementary Fig. S12. This is an illustrative example when only POEs exists for a sample size of 10 000 and the maternal POE effect size is 0.75. These results are indicative of all null simulation settings which show the test was well calibrated under the null when the only effects were parent-of-origin-dependent. Under alternative simulations with a GxE interaction effect, our *post hoc* test had the power to differentiate interaction effects from POEs (Supplementary Figs S13 and S14). Power is increasing with increasing number of phenotypes with non-null interaction effects, sample size, strength of interaction effect, and generally, pairwise trait correlation.

### 3.4 Applied data analysis

**Figure 4:**
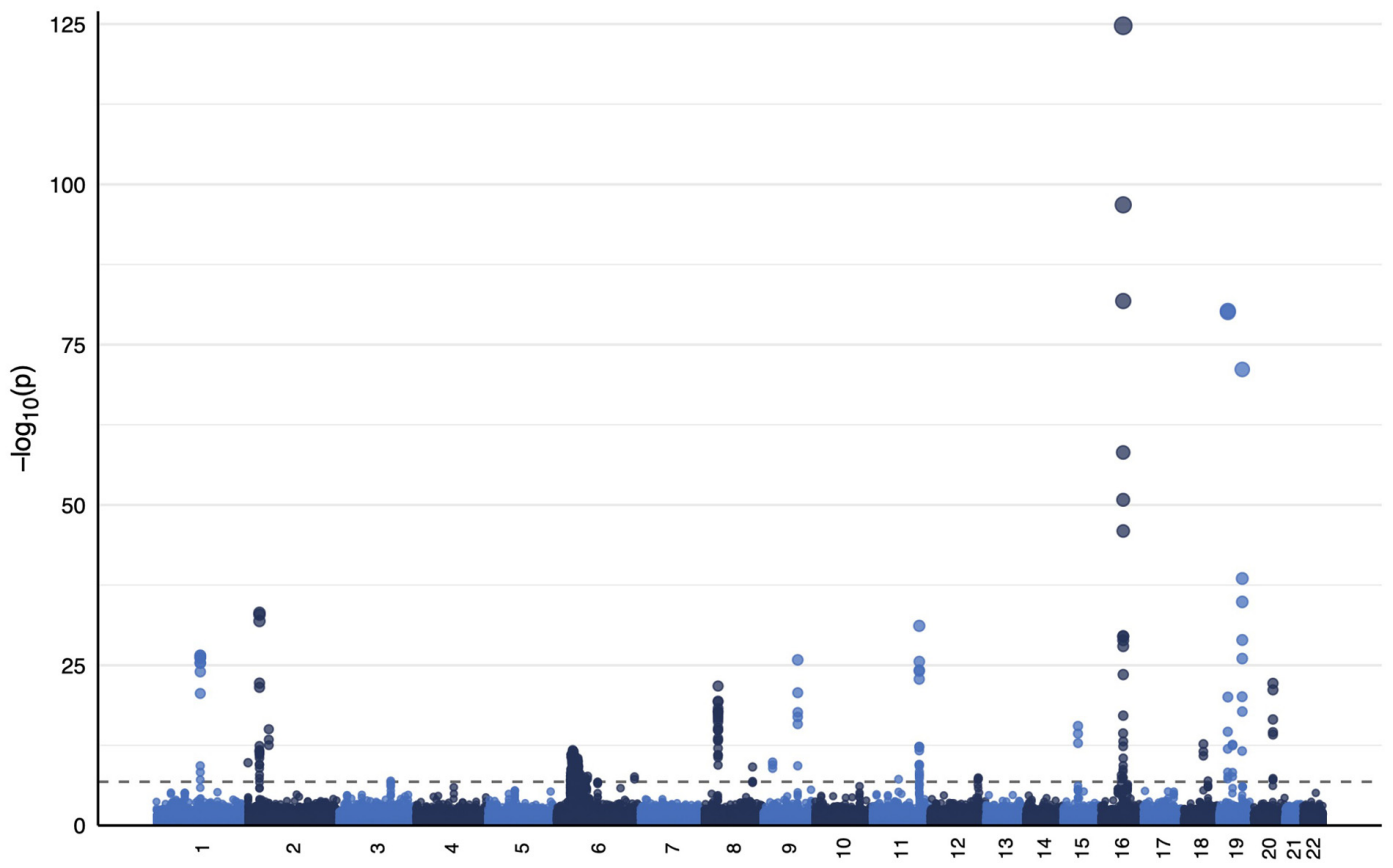
Manhattan plot of parent-of-origin effects analysis using POIROT with BMI, HDL cholesterol, and LDL cholesterol phenotypes from the UK Biobank. The dashed line represents Bonferroni-adjusted genome-wide significance of 1.5 × 10^−7^. BMI, body mass index; HDL, high-density lipoprotein, LDL; low-density lipoprotein.

We applied our method for detecting POEs to genotype and multivariate phenotype data of 292 779 individuals of European ancestry from the UK Biobank. Raw quantitative phenotype measures of interest were BMI, HDL cholesterol, and LDL direct cholesterol. Phenotypes were appropriately adjusted for the effects of genotype array, PCs, sex, age, and smoking status. For the 330 801 variants considered, the average computation time per POIROT test was 22.53 s. Analysis was run with parallel computation with the genome segmented into 793 blocks with a maximum block runtime of 4.7 h (681 variants). We identified a total of 338 variants with POIROT p-values falling below the Bonferroni-adjusted genome-wide significance threshold of 1.5 × 10^−7^ (Supplementary Table S4). These suggestive POE variants are shown in the Manhattan plot in Fig. 4.

We also saw a significant positive normalized enrichment score (nominal *P* < .001) from the GSEA follow-up test, indicating that variants within 500 kb of imprinted or predicted-imprinted genes tended to lie at the top of our list ranked by increasing POIROT *P*-value. We next applied our *post hoc* test to these 338 identified variants to evaluate whether any demonstrated general interaction effects and observed that approximately two-thirds (230) had *P* > .05/338 and failed to reject the null of a POE. We similarly applied the univariate test for POEs genome-wide using each individual phenotype separately. Results are provided in Supplementary Figs S15–S17.

We report on the 41 variants identified by POIROT as potential POE loci that were not identified by any of the three univariate tests for POEs and further were not significantly demonstrating general interaction effects based on our *post hoc* test. These 41 variants thus represent the strongest evidence for novel POE effect(s) in our analysis. Among them, we highlight one exonic variant (Affx-20090007, POIROT *P* = 9.7 × 10^−16^) and one intronic variant (rs41360247, POIROT *P* = 3.0 × 10^−13^) on chromosome 2 for gene *ABCG8*. Polymorphisms in this gene have previously been associated with direct LDL in UKB samples (Klimentidis et al. 2020; Barton et al. 2021). Variants within this gene have additionally been associated with cholesterol phenotypes in analyses outside of the UK Biobank dataset (Willer et al. 2013). Of particular note, *ABCG8* has been shown by prior research to be a high-confidence gene for maternal imprinting (Luedi et al. 2007). We also wish to highlight variants identified by POIROT around the gene *APOB* on chromosome 2. Of 14 POIROT-identified variants mapping to this gene, two failed to show evidence of significant interaction effects by our *post hoc* test [rs550619 (intronic, POIROT *P* = 3.1 × 10^−10^), rs74629722 (intergenic, POIROT *P* = 3.3 × 10^−10^)]. In particular, rs550619 lies 3299 bp from a previously published POE variant for BMI (rs1367117) (Hochner et al. 2015) and has significant GWAS associations with direct LDL levels and total cholesterol phenotypes (Klimentidis et al. 2020; Barton et al. 2021). Neither of these variants were identified for any of the three tested phenotypes using the existing univariate approach to detect POEs.

## 4 Discussion

In this article, we introduce a multivariate method, POIROT, for identifying common variants exhibiting POEs on one or more quantitative phenotypes in unrelated subjects. This work is motivated dually by the widespread evidence of pleiotropy in the genetics literature, as well as the limited statistical options for detecting POEs in unrelated cohorts. Our proposed method is an inherently simple statistical test of whether the phenotypic covariance matrix of heterozygotes is equal to that of homozygotes at a given locus. It represents a multivariate extension of the POE test of a single continuous phenotype proposed by Hoggart et al. (2014). It allows for appropriate adjustment for the effects of important covariates on the phenotypes under study and is also computationally efficient for application to biobank-scale datasets (Supplementary Tables S2 and S3). The R code for implementing POIROT is publicly available (see Data availability).

Through simulations, we demonstrate POIROT achieves appropriate type I error under the null. It further displays superior power to detect POEs than the competing univariate approach under most settings. Our method is indeed robust to non-normality of phenotypes across several simulation scenarios. We further applied our method to real GWAS data on white individuals of European ancestry from the UKB. In this analysis, we considered BMI as well as HDL and direct LDL cholesterol as potential imprinted phenotypes. The analysis revealed 338 variants meeting the stringent genome-wide significance threshold. Of these, 41 of may warrant particular focus in future investigations. They were not identified by the existing univariate approach to detect POEs and did not show evidence of significant gene–gene or gene–environment interaction effects using our proposed *post hoc* test. Two of these variants map to gene *ABCG8*, a gene with high confidence of maternal imprinting in humans based on previously published work, and another lies nearby a known POE variant for BMI in the gene *APOB*.

While the results presented here are promising for the utility of our proposed multivariate method for POE detection in practice, there are inherent limitations that we must address. Firstly, we propose POIROT as a method to detect SNPs wherein the effect of the variant allele in offspring differs according to which parent transmitted it. We do not evaluate the ability of our method to detect other trans-generational effects that may appear as imprinting effects at surface evaluation (Connolly and Heron 2015). Furthermore, we acknowledge that our method to detect POEs by evaluation of differing phenotypic covariance matrices by genotype groups may lead to false positive identifications at loci where gene–environment or gene–gene interaction effects exist. We have proposed a two-stage screening procedure to combat this: first by implementing POIROT as described, and second, by following up with our proposed test to distinguish which findings may be the result of more general interaction effects. We also note if a trans-generational effect exists by which, for example, the maternal genotype is affecting the offspring phenotype in a manner that is not completely independent of offspring genotype (in other words, there are maternal–fetal genotype interaction effects), we do believe we would be able to detect these in our *post hoc* test for interaction effects.

POIROT is a variance/covariance-based test for detecting POEs applicable to large population samples where allelic parental origin is unknown. If parental genetic information is known (i.e. through collection of parent-offspring trios), then it is well-established that variance-based tests within the offspring are often considerably less powerful than mean-based tests (like those described in the Section 1) that leverage allelic parental origin and look for differences in phenotypic means between heterozygous offspring with maternally- versus paternally inherited effect alleles (Struchalin et al. 2010). We performed additional simulations comparing the power of the two strategies at different sample sizes. Specifically, we simulated parent-offspring trio genotype data, restricted samples for analysis to include only heterozygous offspring, and tested for mean-based differences in phenotypes between offspring who inherited the variant alleles maternally versus those who inherited it paternally via one-way MANOVA. We assumed 2 out of 3, 6, or 10 phenotypes harbored a POE. We generally found that variance/covariance methods require ∼13 times as many observations as familial mean-based tests for equivalent power. The trio-based simulations assumed full knowledge of parental transmission of the variant allele in heterozygous offspring, when in reality, parent-of-origin may be ambiguous in certain cases. For details, please see Supplementary Fig. S18. Thus, if family-based data are available, we recommend the use of mean-based tests for POE detection rather than variance-based tests. For population studies, variance-based tests remain the only option for POE analysis.

There are several avenues we are interested in pursuing to extend the work presented here. Rather than testing genome-wide variants, implementation of a two-stage screening procedure may mitigate the multiple testing burden. In the first stage, we propose to perform a standard GWAS for marginal (not parent-of-origin dependent) variant associations that considers multiple traits jointly. We restrict consideration to marginal association tests that are orthogonal to POIROT and thus provide complementary information. We can then efficiently test a smaller subset of top SNPs identified from the first stage for POEs. Another limitation we acknowledge is the requirement of continuous phenotypes. We are interested in the possible extension of our approach to accommodate dichotomous multivariate traits. One potential solution would be to use liability-threshold models (Hujoel et al. 2020) that can effectively transform a binary outcome into a continuous-valued posterior mean genetic liability.

## Supplementary Material

btad199_Supplementary_Data

## Data Availability

The code for implementing this method in R is publicly available at https://github.com/staylorhead/POIROT-POE.
