# Supplementary material for: POIROT: a powerful test for parent-of-origin effects in unrelated samples leveraging multiple phenotypes
Source: Bioinformatics. 2023 Apr 17;39(4):btad199. doi: 10.1093/bioinformatics/btad199 (PMC10148680; doi:10.1093/bioinformatics/btad199)
Supplement: btad199_Supplementary_Data [file btad199_supplementary_data.zip › POIROT_supplemental_figs.docx]

**SUPPLEMENTAL FIGURES**

**Supplemental Figure 1.** Histogram of example simulated non-normal phenotypes assuming skewness = 2 and excess kurtosis = 2. Data shown here corresponds to a sample size of 5,000 for a single phenotype with no parent-of-origin effects.

**Supplemental Figure 2.** Power of POIROT to identify POEs assuming *K* = 3, 6, or 10 normal phenotypes (horizontal panels) compared to univariate test. We assume either 1, 2, or 3 of the phenotypes harbor POEs at the locus with varying magnitude of $\beta_{Mk}$ (vertical panels). We performed 5,000 simulations for each scenario. We calculated power at significance level 0.005 for our multi-trait test and 0.005/*K* (Bonferroni correction) and 0.005/*K_eff_* for the univariate test, where *K_eff_* is the number of PCs needed to explain 90% phenotypic variation. We assume MAF = 0.25 and sample size = 5,000. Abbreviations: POE, parent-of-origin effect; MAF, minor allele frequency; PCs, principal components.

**Supplemental Figure 3.** Power of POIROT to identify POEs assuming *K* = 3, 6, or 10 normal phenotypes (horizontal panels) compared to univariate test. We assume either 1, 2, or 3 of the phenotypes harbor POEs at the locus with varying magnitude of $\beta_{Mk}$ (vertical panels). We performed 5,000 simulations for each scenario. We calculated power at significance level 0.005 for our multi-trait test and 0.005/*K* (Bonferroni correction) and 0.005/*K_eff_* for the univariate test, where *K_eff_* is the number of PCs needed to explain 90% phenotypic variation. We assume MAF = 0.25 and sample size = 10,000. Abbreviations: POE, parent-of-origin effect; MAF, minor allele frequency; PCs, principal components.

**Supplemental Figure 4.** Power of POIROT to identify POEs assuming *K* = 3, 6, or 10 normal phenotypes (horizontal panels) compared to univariate test. We assume either 1, 2, or 3 of the phenotypes harbor POEs at the locus with varying magnitude of $\beta_{Mk}$ (vertical panels). We performed 5,000 simulations for each scenario. We calculated power at significance level 5×10^-4^ for our multi-trait test and 5×10^-4^/*K* (Bonferroni correction) and 5×10^-4^/*K_eff_* for the univariate test, where *K_eff_* is the number of PCs needed to explain 90% phenotypic variation. We assume MAF = 0.25 and sample size = 5,000. Abbreviations: POE, parent-of-origin effect; MAF, minor allele frequency; PCs, principal components.

**Supplemental Figure 5.** Power of POIROT to identify POEs assuming *K* = 3, 6, or 10 normal phenotypes (horizontal panels) compared to univariate test. We assume either 1, 2, or 3 of the phenotypes harbor POEs at the locus with varying magnitude of $\beta_{Mk}$ (vertical panels). We performed 5,000 simulations for each scenario. We calculated power at significance level 5×10^-4^ for our multi-trait test and 5×10^-4^/*K* (Bonferroni correction) and 5×10^-4^/*K_eff_* for the univariate test, where *K_eff_* is the number of PCs needed to explain 90% phenotypic variation. We assume MAF = 0.25 and sample size = 10,000. Abbreviations: POE, parent-of-origin effect; MAF, minor allele frequency; PCs, principal components.

**Supplemental Figure 6.** Power of POIROT to identify POEs assuming *K* = 3, 6, or 10 non-normal phenotypes (horizontal panels) compared to univariate test. We assume either 1, 2, or 3 of the phenotypes harbor POEs at the locus with varying magnitude of $\beta_{Mk}$ (vertical panels). We performed 5,000 simulations for each scenario. We calculated power at significance level 0.005 for our multi-trait test and 0.005/*K* (Bonferroni correction) and 0.005/*K_eff_* for the univariate test, where *K_eff_* is the number of PCs needed to explain 90% phenotypic variation. We assume MAF = 0.25 and sample size = 5,000. Abbreviations: POE, parent-of-origin effect; MAF, minor allele frequency; PCs, principal components.

**Supplemental Figure 7.** Power of POIROT to identify POEs assuming *K* = 3, 6, or 10 non-normal phenotypes (horizontal panels) compared to univariate test. We assume either 1, 2, or 3 of the phenotypes harbor POEs at the locus with varying magnitude of $\beta_{Mk}$ (vertical panels). We performed 5,000 simulations for each scenario. We calculated power at significance level 0.005 for our multi-trait test and 0.005/*K* (Bonferroni correction) and 0.005/*K_eff_* for the univariate test, where *K_eff_* is the number of PCs needed to explain 90% phenotypic variation. We assume MAF = 0.25 and sample size = 10,000. Abbreviations: POE, parent-of-origin effect; MAF, minor allele frequency; PCs, principal components.

**Supplemental Figure 8.** Power of POIROT to identify POEs assuming *K* = 3, 6, or 10 non-normal phenotypes (horizontal panels) compared to univariate test. We assume either 1, 2, or 3 of the phenotypes harbor POEs at the locus with varying magnitude of $\beta_{Mk}$ (vertical panels). We performed 5,000 simulations for each scenario. We calculated power at significance level 5×10^-4^ for our multi-trait test and 5×10^-4^/*K* (Bonferroni correction) and 5×10^-4^/*K_eff_* for the univariate test, where *K_eff_* is the number of PCs needed to explain 90% phenotypic variation. We assume MAF = 0.25 and sample size = 5,000. Abbreviations: POE, parent-of-origin effect; MAF, minor allele frequency; PCs, principal components.

**Supplemental Figure 9.** Power of POIROT to identify POEs assuming *K* = 3, 6, or 10 non-normal phenotypes (horizontal panels) compared to univariate test. We assume either 1, 2, or 3 of the phenotypes harbor POEs at the locus with varying magnitude of $\beta_{Mk}$ (vertical panels). We performed 5,000 simulations for each scenario. We calculated power at significance level 5×10^-4^ for our multi-trait test and 5×10^-4^/*K* (Bonferroni correction) and 5×10^-4^/*K_eff_* for the univariate test, where *K_eff_* is the number of PCs needed to explain 90% phenotypic variation. We assume MAF = 0.25 and sample size = 10,000. Abbreviations: POE, parent-of-origin effect; MAF, minor allele frequency; PCs, principal components.

**Supplemental Figure 10.** Power of POIROT to identify POEs assuming *K* = 3, 6, or 10 normal phenotypes (horizontal panels) compared to univariate test. We assume either 2 or 3 of the phenotypes harbor POEs at the locus with varying magnitude of $\beta_{Mk}$ (vertical panels). We performed 5,000 simulations for each scenario. We calculated power at significance level 5×10^-4^ for our multi-trait test and 5×10^-4^/*K* (Bonferroni correction) and 5×10^-4^/*K_eff_* for the univariate test, where *K_eff_* is the number of PCs needed to explain 90% phenotypic variation. We assume MAF = 0.25 and sample size = 5,000. Abbreviations: POE, parent-of-origin effect; MAF, minor allele frequency; PCs, principal components.

**Supplemental Figure 11.** Power of POIROT to identify POEs assuming *K* = 3, 6, or 10 normal phenotypes (horizontal panels) compared to univariate test. We assume either 2 or 3 of the phenotypes harbor POEs at the locus with varying magnitude of $\beta_{Mk}$ (vertical panels). We performed 5,000 simulations for each scenario. We calculated power at significance level 5×10^-4^ for our multi-trait test and 5×10^-4^/*K* (Bonferroni correction) and 5×10^-4^/*K_eff_* for the univariate test, where *K_eff_* is the number of PCs needed to explain 90% phenotypic variation. We assume MAF = 0.25 and sample size = 10,000. Abbreviations: POE, parent-of-origin effect; MAF, minor allele frequency; PCs, principal components.

**Supplemental Figure 12.** QQ plots of p-values for proposed post-hoc test for gene-gene or gene-environment interaction effects under the null hypothesis of no interactions effects but under the presence of POEs. Simulations used 10,000 individuals with 3 (left column), 6 (middle column) or 10 (right column) continuous normal phenotypes with medium correlation. MAF is assumed to be 0.25. Horizontal panels depict number of phenotypes with POE (1, 2, or 3) and maternal POE effect size of 0.75. Abbreviations: QQ, quantile-quantile; MAF, minor allele frequency.

**Supplemental Figure 13.** Power of post-hoc test for interaction effects assuming *K* = 3, 6, or 10 normal phenotypes (horizontal panels). We assume either 1, 2, or 3 of the phenotypes harbor gene-environment interaction effects at the locus with varying magnitude of covariate effect size (vertical panels). Color corresponds to proportion of phenotypic variation explained by interaction effects for an affected phenotype. We performed 5,000 simulations for each scenario. We calculated power at significance level 5×10^-4^. We assume MAF = 0.25 and sample size = 5,000.

**Supplemental Figure 14.** Power of post-hoc test for interaction effects assuming *K* = 3, 6, or 10 normal phenotypes (horizontal panels). We assume either 1, 2, or 3 of the phenotypes harbor gene-environment interaction effects at the locus with varying magnitude of covariate effect size (vertical panels). Color corresponds to proportion of phenotypic variation explained by interaction effects for an affected phenotype. We performed 5,000 simulations for each scenario. We calculated power at significance level 5×10^-4^. We assume MAF = 0.25 and sample size = 10,000.


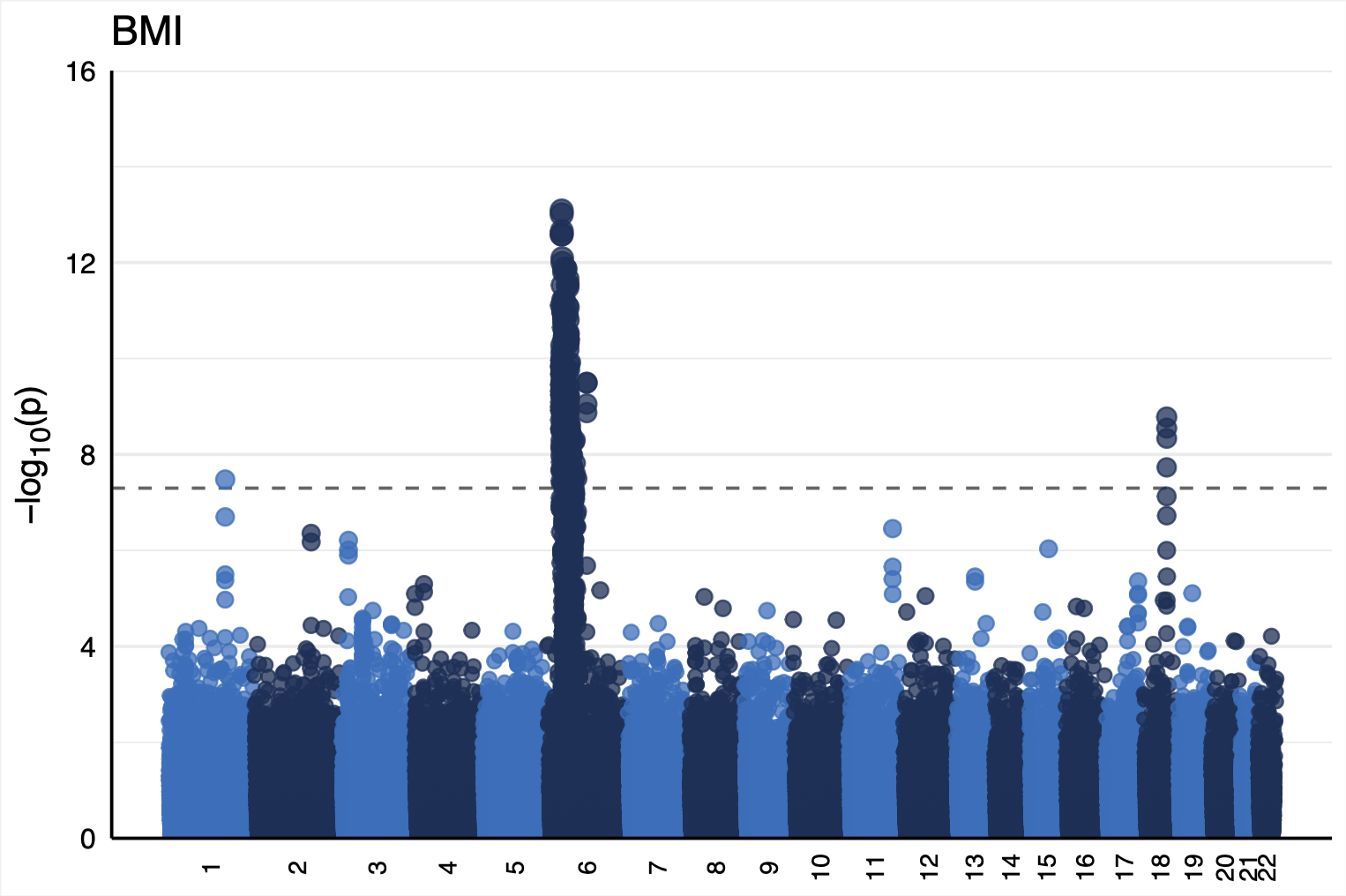


**Supplemental Figure 15.** Manhattan plot of parent-of-origin effects analysis using univariate test with BMI as phenotype from the UK Biobank. The dashed line represents Bonferroni-adjusted genome-wide significance for 330,801 variants and three phenotypes (992,403 tests). Abbreviations: BMI, body mass index.


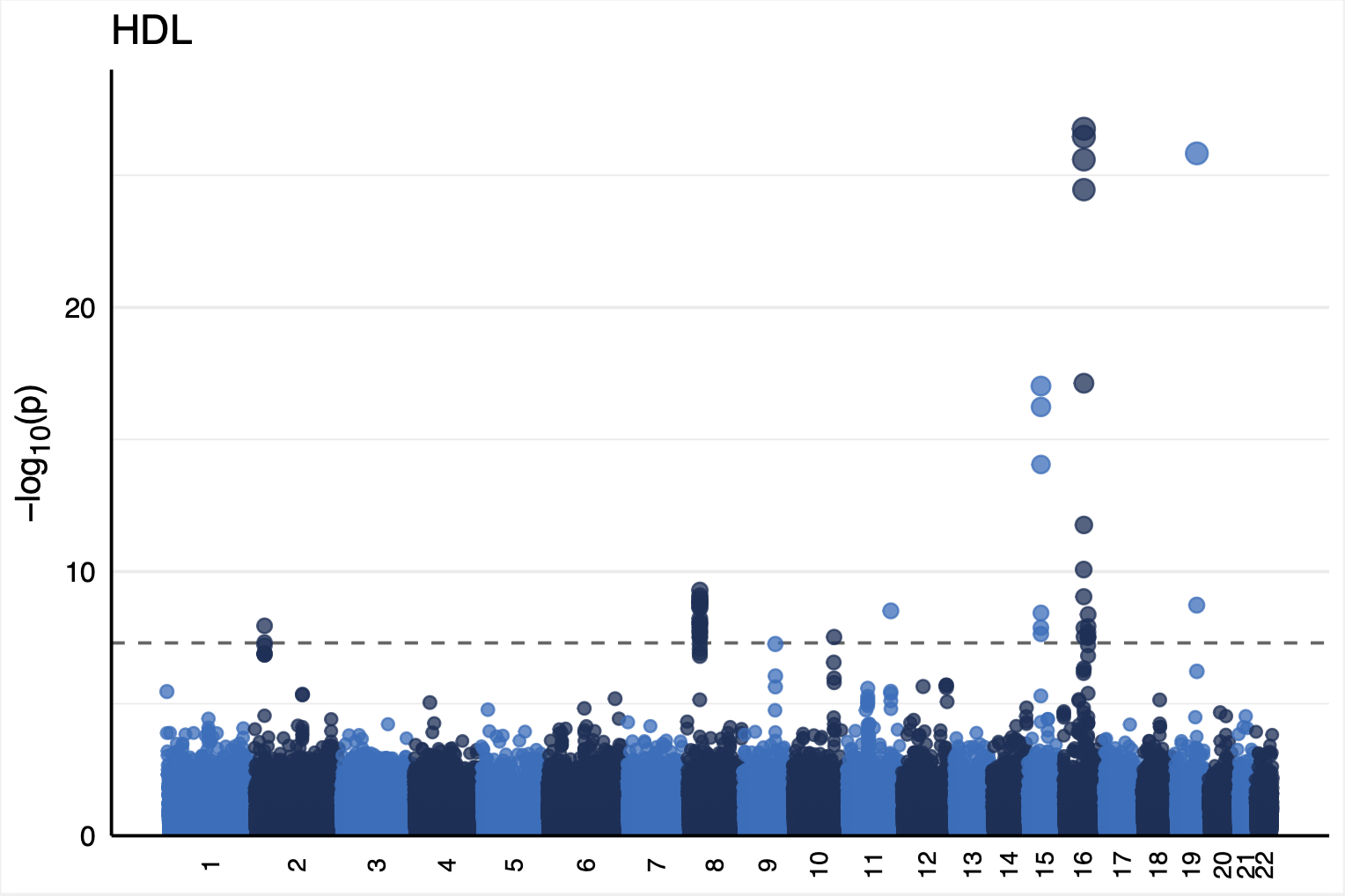


**Supplemental Figure 16.** Manhattan plot of parent-of-origin effects analysis using univariate test with HDL cholesterol as phenotype from the UK Biobank. The dashed line represents Bonferroni-adjusted genome-wide significance for 330,801 variants and three phenotypes (992,403 tests). Abbreviations: HDL, high-density lipoprotein.


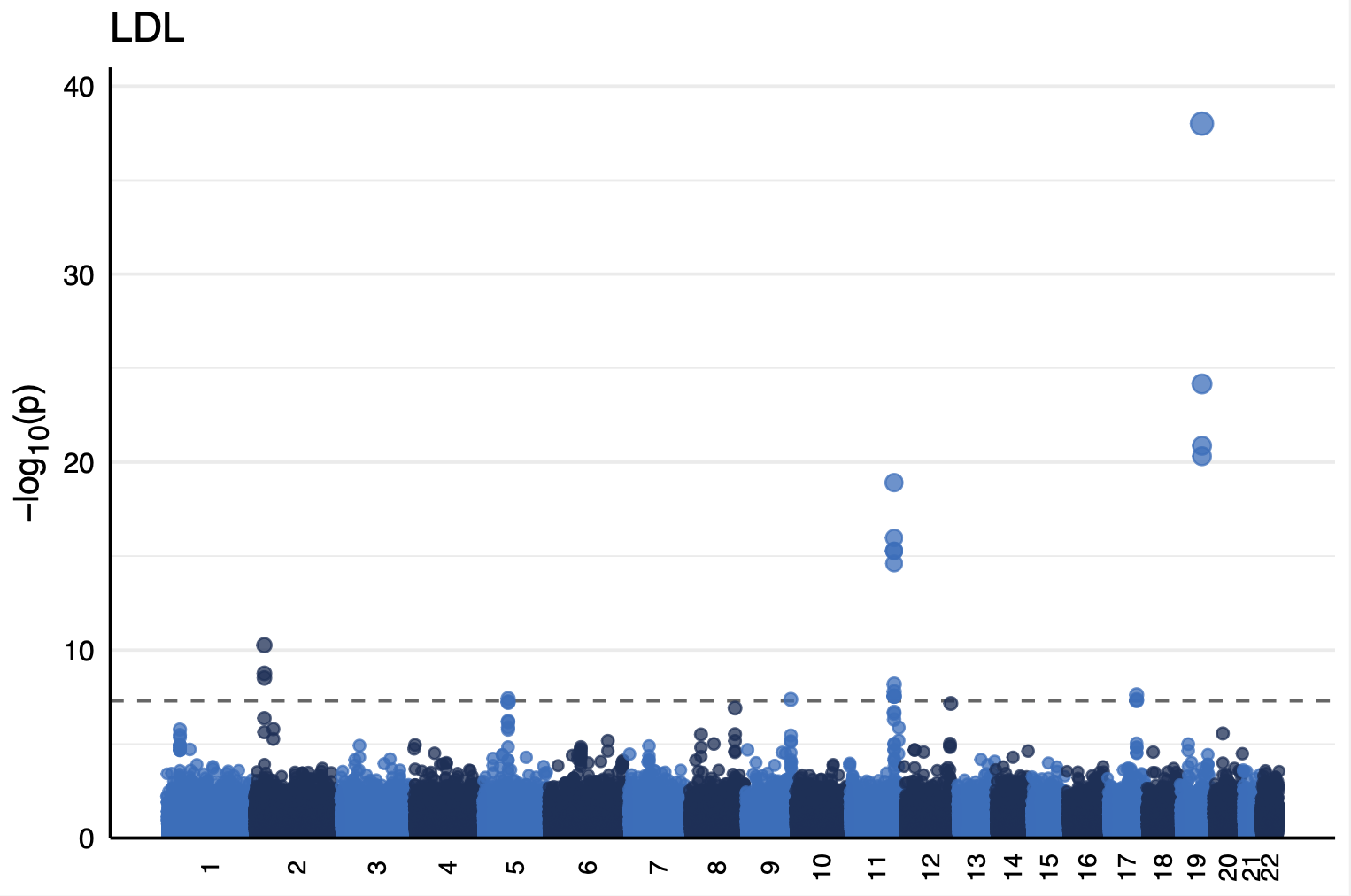


**Supplemental Figure 17.** Manhattan plot of parent-of-origin effects analysis using univariate test with LDL direct cholesterol as phenotype from the UK Biobank. The dashed line represents Bonferroni-adjusted genome-wide significance for 330,801 variants and three phenotypes (992,403 tests). Abbreviations: LDL, low-density lipoprotein.

**Supplemental Figure 18.** Power comparison of POIROT to a family-based approach using trio genotype data. Black line represents the power of POIROT at given sample size of unrelated individuals with no family genotype data (x-axis). Horizontal lines represent power of one-way MANOVA comparing phenotypic means of heterozygous offspring with maternally inherited minor allele versus heterozygous offspring with a paternally inherited minor allele. Green line represents family-based approach power at trio size 500, blue corresponds to trio size 300, and red represents trio size 250. Given trio size *N*, this corresponds to approximately 2*(MAF)(1-MAF)N* heterozygous offspring. Simulation parameters included MAF = 0.25, $\beta_{M}$ = 0.5, normal error distribution, and medium pairwise phenotype correlation. Of the 3, 6, or 10 total phenotypes tested in each analysis, we assumed 2 harbored parent-of-origin effects. Abbreviations: MAF, minor allele frequency.
